# Exosomes Released from Breast Cancer Carcinomas Stimulate Cell Movement

**DOI:** 10.1371/journal.pone.0117495

**Published:** 2015-03-23

**Authors:** Dinari A. Harris, Sajni H. Patel, Marjan Gucek, An Hendrix, Wendy Westbroek, Justin W. Taraska

**Affiliations:** 1 Laboratory of Molecular Biophysics, National Heart Lung and Blood Institute, National Institutes of Health, Bethesda, Maryland, United States of America; 2 Proteomics Core Facility, National Heart Lung and Blood Institute, National Institutes of Health, Bethesda, Maryland, United States of America; 3 Laboratory of Experimental Cancer Research, Department of Radiation Oncology and Experimental Cancer Research, Ghent University Hospital, Ghent, Belgium; 4 Medical Genetics Branch, National Human Genome Research Institute, National Institutes of Health, Bethesda, Maryland, United States of America; Stony Brook University, UNITED STATES

## Abstract

For metastasis to occur cells must communicate with to their local environment to initiate growth and invasion. Exosomes have emerged as an important mediator of cell-to-cell signalling through the transfer of molecules such as mRNAs, microRNAs, and proteins between cells. Exosomes have been proposed to act as regulators of cancer progression. Here, we study the effect of exosomes on cell migration, an important step in metastasis. We performed cell migration assays, endocytosis assays, and exosome proteomic profiling on exosomes released from three breast cancer cell lines that model progressive stages of metastasis. Results from these experiments suggest: (1) exosomes promote cell migration and (2) the signal is stronger from exosomes isolated from cells with higher metastatic potentials; (3) exosomes are endocytosed at the same rate regardless of the cell type; (4) exosomes released from cells show differential enrichment of proteins with unique protein signatures of both identity and abundance. We conclude that breast cancer cells of increasing metastatic potential secrete exosomes with distinct protein signatures that proportionally increase cell movement and suggest that released exosomes could play an active role in metastasis.

## INTRODUCTION

Exosomes are small membrane vesicles (30–100nm) derived from the luminal membranes of multivesicular bodies (MVB) and are released from mammalian cells by exocytosis [1–5]. Along with diffusible signals, such as cytokines, growth factors, and proteases, exosomes mediate short- and long-range cell-to-cell communication by transferring proteins, RNA, and lipids between cells [5–9]. Exosome release occurs under normal physiological conditions and abnormal release of exosomes can arise in diseases such as cancer. The magnitude of exosome release has been linked to tumor invasiveness both *in vitro* and *in vivo* [10,11]. Exosomes are small enough to penetrate into and interact with tissues, and have been shown to promote increased migration and proliferation of tumors [12–14]. Exosomes have also been shown to affect unique stages of tumor progression, including angiogenesis, escape from immune surveillance, extracellular matrix degradation, and metastasis [15–20].

For metastasis to occur, a cell must manipulate its local environment to optimize invasion and growth [21–23]. The molecular steps of metastasis can be divided into 3 stages: (1) loss of adhesion; (2) increased migration; and (3) increased invasion. The metastatic potential of cancer cells is a term given to cancers to classify the level of phenotypic changes that are linked to increased metastatic behaviors [24]. For example, a high metastatic potential correlates with high rates of migration and motility. A subset of specific genes that regulate the tumor microenvironment are positively linked to the increased invasiveness (increased metastatic potential) of the cancer [24–28]. Thus, this classification can be gained from several experimental methods including microarray analysis, gene-expression profiling, and proteomics. A similar signature has been suggested for other signaling components of cancers, including exosomes [29–34].

Here, we examined the effects of exosomes on cell migration, a key step in metastasis. We show that exosomes stimulate cell migration. Furthermore, we show that exosomes induce migration proportional to the metastatic potential of the cell from which the exosomes originated. We then identified and quantified the proteins associated with these exosomes. From this work, we provide the first comprehensive proteomic catalog of exosomes isolated from breast cancers cells of increasing metastatic potentials. Our results support the idea that exosomes are a positive signal for cell motility and growth. This signal is stronger in exosomes from cells with higher metastatic potentials [35]. Our work suggests a role for exosomes in accelerating cancer progression and identifies new biomarkers that could be used as therapeutic targets or indicators of metastasis.

## RESULTS

**Fig 1:**
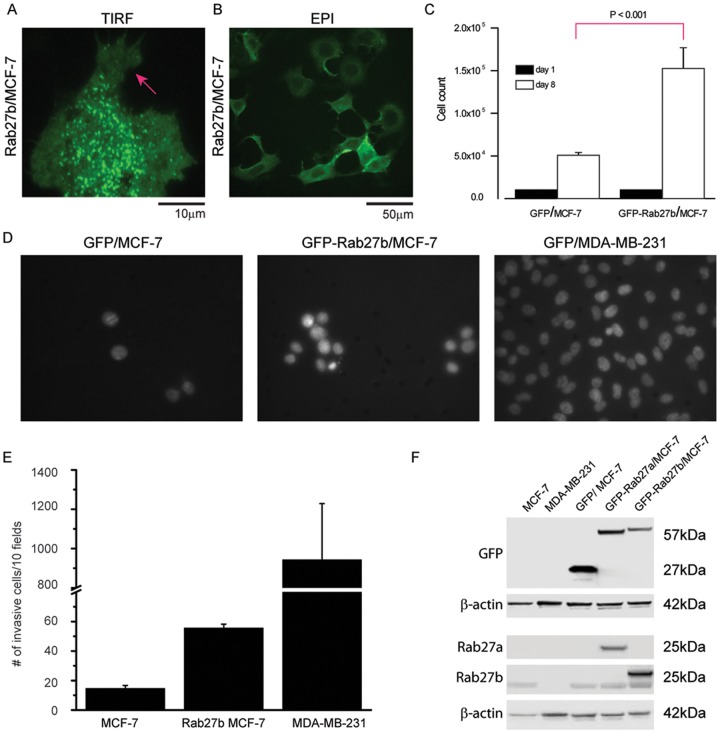
Characterization of invasiveness associated with non-metastatic and metastatic breast cancer cell lines. (A) Punctate expression pattern and cellular extensions of MCF-7 cells stably transfected with GFP-Rab27b-expressing plasmids. (B) Cells overexpression GFP-Rab27b formed cellular extensions with peripherally-localized GFP-Rab27b, and a spreading morphology on collagen-coated coverslips. (C) Measurement of cell proliferation of MCF-7 cells stably expression GFP or GFP-Rab27b. The same number of cells was plated in triplicate into 10 cm dishes on day 1 and the total number of cells was counted on day 8. (D) Matrigel invasion assay with MCF-7 GFP, MCF-7 Rab27b GFP, and MDA-MB-231cells. 10^5^ cells were seeded in serum free media on a Matrigel-coated filter and their migration toward medium containing serum was quantified by microscopic evaluation. MCF-7 cells stably overexpressing GFP-Rab27b showed significant increased invasion into matrigel compared to MCF-7 GFP cells (mean = 55.7 vs. 14.7 cells, *P<0*.*0001*). The well-characterized invasive MDA-MB-231 cell line showed massive invasion into matrigel, which has been described previously [62]. (E) The mean total number of invading cells from 10 different fields is shown *P* values were calculated using two-sided Student *t* tests. Statistically significant *P* values are indicated (n = 3); (F) Western blot analysis of protein extracts from MCF-7, MDA-MB-231, MCF-7 /GFP, MCF-7/GFP-RAB27a, and MCF-7/GFP-RAB27b probed with antibodies to GFP, RAB27a, and RAB27b. An anti-HRP conjugated β-actin antibody was used as a protein loading control. The established MCF-7 RAB27b GFP cell line showed over-expression of GFP-RAB27b demonstrated with an anti-GFP antibody, RAB27b antibody, and RAB27a antibody. Lysates of previously described cell lines (MCF-7 GFP, MCF-7 RAB27a GFP) were included as controls to show specificity of the antibodies [37].

To examine the role of released exosomes on cell motility, we first isolated exosomes from cultured cells that represent different metastatic potentials. We chose MCF-7 and MDA-MB-231 cells, two commonly used breast cancer cell lines [26,36]. MCF-7 cells are tumorigenic but non-metastatic and represent the lowest metastatic potential in this study. MDA-MB-231 cells are highly metastatic, with altered adhesion and motility properties and thus have the highest metastatic potential in this study. To develop a model cell line with intermediate metastatic potential we created an MCF-7 cell line that stably over-expresses GFP-tagged Rab27b. Increased expression of Rab27b has been shown to promote G1 to S phase cell cycle transition, proliferation, and invasiveness of cells in culture. Rab27b also has been shown to promote invasive tumor growth in mouse xenograph models. When we plated MCF-7 cells that overexpress GFP-Rab27b on type I collagen coverslips, we observe four distinct changes in these cells. 1) They exhibited a more extended morphology characteristic of more metastatic cells. 2) They showed increased motility. 3) Rab27b-GFP exhibited cell peripheral localization (Fig. 1A-B), and 4) the cells exhibited a (~3-fold) increase in cell proliferation over control cells transfected with GFP at limiting (0.5%) serum concentrations (Fig. 1C) in agreement with previous work [37]. Rab27b levels were additionally determined in the three cell lines (MCF-7; Rab27b/MCF-7; and MDA-MB-231) by western blotting (Fig. 1F). Thus, MCF-7 cells that stably overexpress Rab27b (referred to as Rab27b cells) represent a tumorigenic and transformed line of moderate metastatic potential in this study. The levels of metastasis associated with each breast cancer cell line was further confirmed and validated with a matrigel cell invasion assay (Fig. 1D-E).

**Fig 2:**
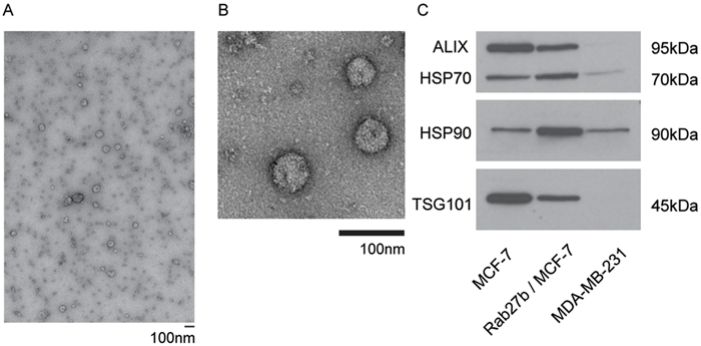
Characterization of exosomes. (A) Large-field view of electron micrograph of exosomes released from the human breast cancer cell line MCF-7 and stained with uranyl acetate. Scale bar, 100nm; (B) Magnified view of electron micrograph of exosomes released from the human breast cancer cell line MCF-7. Scale bars are 100nm. (C) Western blot analysis of exosomal proteins extracted from MCF-7, MCF-7/Rab27b, and MDA-MB-231.

**Fig 3:**
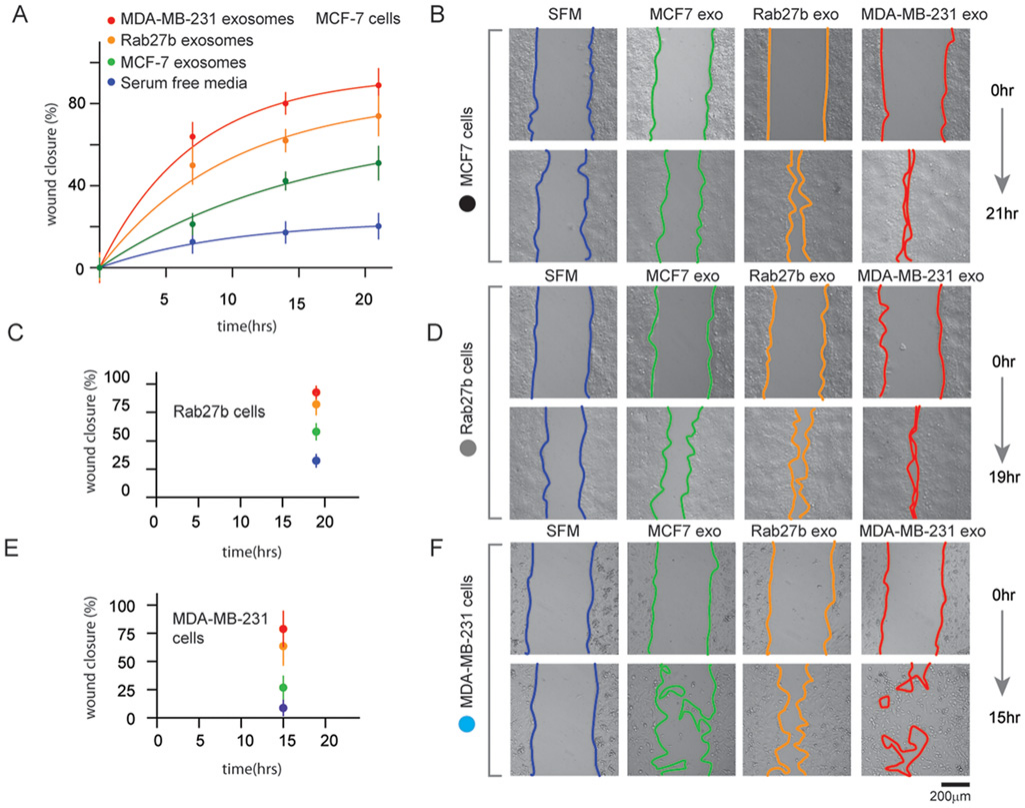
Tumor-derived exosomes increase breast cell motility. (A) Time course of cell migration wound-healing assays using MCF-7 breast cancer cells as the “recipient” cell line with the three “donor” exosome preparations. Serum-free media was used as a control. Each point on the assay represents three independent experiments at 21 hours. (B) Representative images from the wound healing experiments. Quantification (C) and images (D) of wound-healing assay for MCF7/Rab27b cells in the presence of the three exosome preparations and serum-free media after 19 hours. Quantification (C) and images (D) of wound-healing assay for MDA-MB-231 cells in the presence of the three exosome preparations and serum-free media after 15 hours. Errors were calculated from wound closure at each time point and normalized to the wound closure at the initial time point (0 hour). Experiments were repeated two additional times to verify results.

Exosomes purified from these three cells by serial ultra-centrifugation [38] were observed by transmission electron microscopy to be small (50–120nm) spherical vesicles (Fig. 2A-B). To ensure that we isolated exosomes from our preparations, we conducted Western blotting to confirm the presence of several common exosome/vesicle markers, including TSG101, ALIX, HSP70, HSP90 (Fig. 2C). The presence of these markers is consistent with purified exosome samples. Additionally, we further analyzed (in triplicate) our exosome preparations using nanoparticle tracking analysis (NTA) which measures particles (*eg*. microvesicles). Based on these measurements and the shoulder associated with the major distribution peak (Figure A-C in S1 Fig.), it is likely that our exosome preparations isolated from MDA-MB-231 (and to a lesser extent also for MCF-7/Rab27b) contain a heterogeneous mixture of exosome and microvesicles consistent with other high speed ultracentrifugation protocols [39]. This finding is consistent with the work which suggested that several diverse population of vesicles (including exosomes, microvesicles, ectosomes, membrane particles, exosome-like vesicles, and apoptotic vesicles) are present in many exosome preparations obtained by differential ultracentrifugation [39,40]. To test the effect of these vesicles on cell motility, we added the same concentration of exosomes isolated from the three different “donor” breast cancer cells types (MCF-7, Rab27b, MDA-MB-231) to “recipient” cells of the same or different identity from which the exosomes were originally derived. Migration was measured in a standard wound healing assay. In this assay, cells were plated into two zones with a space separating each population. The decrease in the wound caused by the migration of cells over time was then quantified (Fig. 3). We observe a substantial and reproducible wound closure (or increase in cell motility) in cells incubated with exosomes isolated from all three donor cancer cell types compared to cells incubated in control media (Serum-free media (SFM), with PBS only). Exosomes isolated from the moderate and highly metastatic cells (Rab27b and MDA-MB-231) induced increases in cell migration in all three recipient cell lines (Fig. 3B, D, E). Interestingly, exosomes isolated from the cells with the highest metastatic potential (MDA-MB-231) induced the largest increase in motility, followed by Rab27b cell line (Fig. 3C-F). The effect on cell migration induced by the exosomes purified from the non-metastatic MCF-7 cells was modest compared to controls (Fig. 3A-B). These data show that the effect of exosomes on migration is linked to the underlying metastatic potential of the donor cells. Interestingly, exosomes from highly metastatic cells induced migration to a degree that depended on the metastatic potential of the donor cancer cell type. In short, exosomes derived from the donor metastatic lines (Rab27b and MDA-MB-231) promoted cell mobility faster and/or to a further extent than exosomes isolated from the non-metastatic cell line (MCF-7) or control media. The migration phenotype in the moderate metastatic line (Rab27b) occurred faster than the non-metastatic line (MCF-7) and the MDA-MB-231 cells healed the fastest overall.

**Fig 4:**
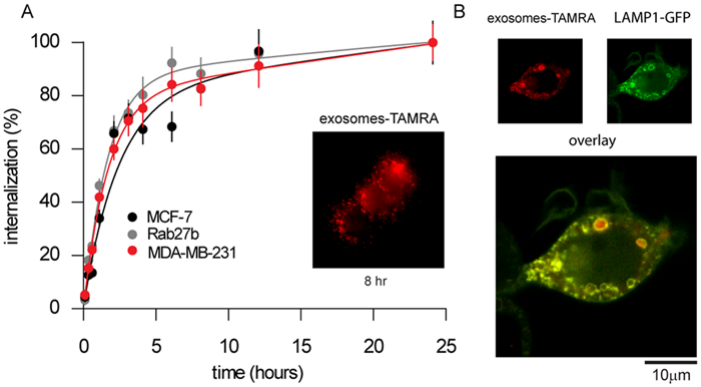
Endocytosis of exosomes in breast cancer cells. (A) Time-course curve of exosome uptake (endocytosis) by determining fluorescent intensity of TAMRA-labeled exosomes from “donor” cancer cells at specific times. Inset shows a MCF-7 “recipient” cells incubated with TAMRA-labeled MCF-7 “donor” exosomes at 8 hours. Errors were calculated from fluorescence intensity of cells (n = 50), at each time point, normalized to the intensity at the final time point (24 hours). (B) Colocalization of TAMRA-labeled exosomes added to MCF-7 cells transiently transfected with LAMP1-GFP. Scale bar is 10μm.

Because our data suggests that the uptake of exosomes enhances cell migration, we next asked if the effect of exosomes was due to a differential rate of endocytosis of the exosomes by the recipient cells. To monitor endocytosis of exosomes, recipient cells were exposed to fluorescently-labeled exosomes derived from the same donor cell types, followed by extensive PBS and acid washing to remove extracellular membrane bound exosomes. The increase in cytosolic fluorescence which relates to the quantity of exocytosis taken up by the recipient cells was monitored with epi-fluorescence during the treatment [41]. The rate of exosome uptake (Fig. 4A) was calculated by determining the average fluorescence intensity in the cytoplasm as a function of time. In these experiments, small fluorescent puncta were clearly visible inside cells within 5 minutes of exposure and more than 50% maximal fluorescent uptake occurred after 3 hours of exposure. The fluorescence intensity reached a maximum by 8 hours, suggesting that exosome uptake had saturated (Fig. 4A inset). The subcellular distribution of exosomes was further studied by colocalization with the lysosomal marker LAMP1 (lysosomal-associated membrane protein 1). Colocalization of LAMP1-GFP and TAMRA-labeled exosomes indicated that some of the exosomes were sorted to lysosomes (Fig. 4B). In all three cell lines, however, endocytosis of exosomes showed little difference in overall quantity or rate. From these data we conclude that the differences in motility effect of exosomes derived from donor cancer cells on target cells is not due to differences in the rate of endocytosis but instead is likely related to the distinct molecular composition of the exosomes.

**Fig 5:**
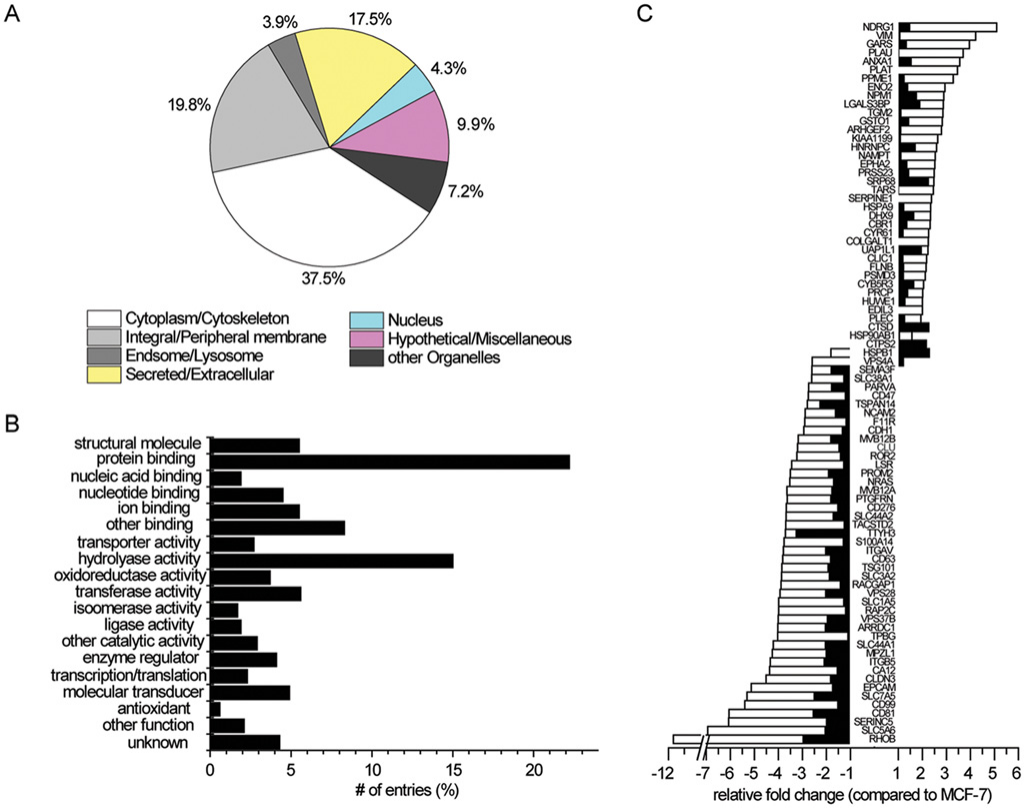
Cytolocalization, molecular function, and the relative abundance of proteins identified in tumor-derived exosomes. (A) Cellular localization of exosome proteins. Relative subcellular distribution of proteins identified in the exosomes isolated from the breast cancer cell line, MCF-7. Classification of the subcellular location of the proteins was based on the information provided by the UniProtKB/SwissProt database. (B) GO annotation of tumor-derived proteins. Proteins identified were allocated to different molecular function categories defined by the GO consortium. Relative protein levels of exosomes isolated from MCF-7 are indicated by the bars. Two independent biological replicates from the breast cancer cells type, MCF-7 were used for MS analysis. (C) iTRAQ analysis: Differential expression profile for metastatic tumor-derived exosome proteins in MDA-MB-231 and Rab27b-transformed MCF-7 cells compared to non-invasive breast cancer cells (MCF-7). Filled and open bars represent fold change in protein expression in Rab27b and MDA-MB-231 compared to MCF-7, respectively. Two independent biological replicates from the 3 cells types (MCF-7; Rab27b; MDA-MB-231) were used for iTRAQ analysis. The raw data give absolute expression levels of the various proteins (identity) and proteins levels (abundance) in all 3 cells lines. The average of proteins from the two independent samples from Rab27b and MDA-MB-231 lines was determined and compared to that the average of two samples from MCF-7 and the ratio for differential expression was determined.

Because we found that exosomes from highly metastatic cells induce greater motility, we next identified the exosomal proteome from the three different donor cancer cell types. The proteins associated with tumor-derived exosomes were identified by nano-LC-MS analysis of peptides obtained by in-gel digestion of SDS-PAGE gel bands. We identified a total of 513 distinct proteins in the MCF-7 cell exosomes [42] (S1 Table). As expected, many membrane-associated proteins were identified as well as many proteins associated with other diverse cellular locations (Fig. 5A) and functions (Fig. 5B). Analysis of the subcellular localization of the identified proteins suggested that the largest class was cytosolic proteins (37.5%). Significant portions were integral and peripheral membrane proteins (19.8%), proteins known to be located extracellularly (17.5%). A smaller fraction of endosomal/lysosomal proteins (3.9%) and proteins assigned to the Golgi apparatus, ER, or mitochondria (other organelles; 7.2%) were identified. The smallest fraction was nuclear proteins (4.3%). To obtain a functional overview of the exosomal protein compositions, we annotated our proteomics into functional categories (Fig. 5B). A total 491 of the 513 proteins identified could be assigned to defined molecular functions. Protein binding (22%) was significantly represented in MCF-7 cell-derived exosomes, followed by an abundant fraction of proteins with hydrolase activity (15%). The remaining proteins were distributed over several general groups including structural proteins (~6%), ion binding proteins (~6%), oxidoreductases (~4%), transferases (~6%), enzyme regulators (~4%), and molecular transducers (~5%). These data suggest that the tumor-derived exosomes contain proteins with extremely diverse functions and cellular origins.

The function of exosomes is dependent on the cargo they carry, which is dependent on the cell type they were derived [43]. Thus, differences in expression of specific exosomal proteins could be related to the increases in migration induced by metastatic cell-derived exosomes (Rab27b and MDA-MB-231). Next, we quantified the relative amount and identity of exosomal proteins from our breast carcinoma cell lines [24] and performed iTRAQ-based quantitative proteomic analysis. Exosomal proteins were digested, labeled, and analyzed using LC-MS/MS. We accepted protein identities based on at least 2 peptides with a probability higher than 95% and minimal total protein identification probability of 99%. This yielded the identification of 1,312 proteins (S2 Table). To compare the proteome of exosomes from the three donor breast cancer cell lines, iTRAQ-labeled peptides from all three tumor-derived exosomes preparations were quantified [44]. Two biological replicates for each sample were included for reproducibility. After manual inspection of quantified peptides, differently expressed proteins were identified. Fig. 5C shows the proteins that were differentially expressed and their relative levels compared to MCF-7 cells. This analysis identified 85 differentially expressed proteins (~2-fold increase or decrease in relative expression compared to protein expression in MCF-F cells) across the three cell lines. Surprisingly, we observed that the exosomes from the Rab27b cell line show the same expression trends as MDA-MB-231 cells. Specifically, the levels the Rab27b cell line exosomes varied from protein to protein but were in general less abundant than those in the MDA-MB-231 exosomes. The overall expression signature, however, of the Rab27b exosomes shows a similar profile to the MDA-MB-231 exosomes (Fig. 5C). Of the 38 up-regulated proteins, 30 proteins (79%) have been shown to be involved in metastasis and invasion. The overexpression of these 30 proteins has been shown to be correlated with accelerated tumor growth, invasion, and poor prognosis, and poor outcome in patients [24,26,27].

## DISCUSSION

Metastasis requires breaking of cell-to-cell and cell-to-matrix interactions. This allows cells to acquire a mobile phenotype [10,11,45]. In this study, we examined the role of exosomes in cancer cell motility and the progression towards metastasis. We tested the effects of exosomes from cells of varying invasiveness on cell migration. In migration assays, we show that the ability of exosomes to promote motility correlated with the metastatic potential of the cells which released the exosomes. We further tested whether increases in migration induced by exosomes were the result of changes in endocytosis of exosomes. Endocytosis assays showed no differences in exosome uptake.

**Fig 6:**
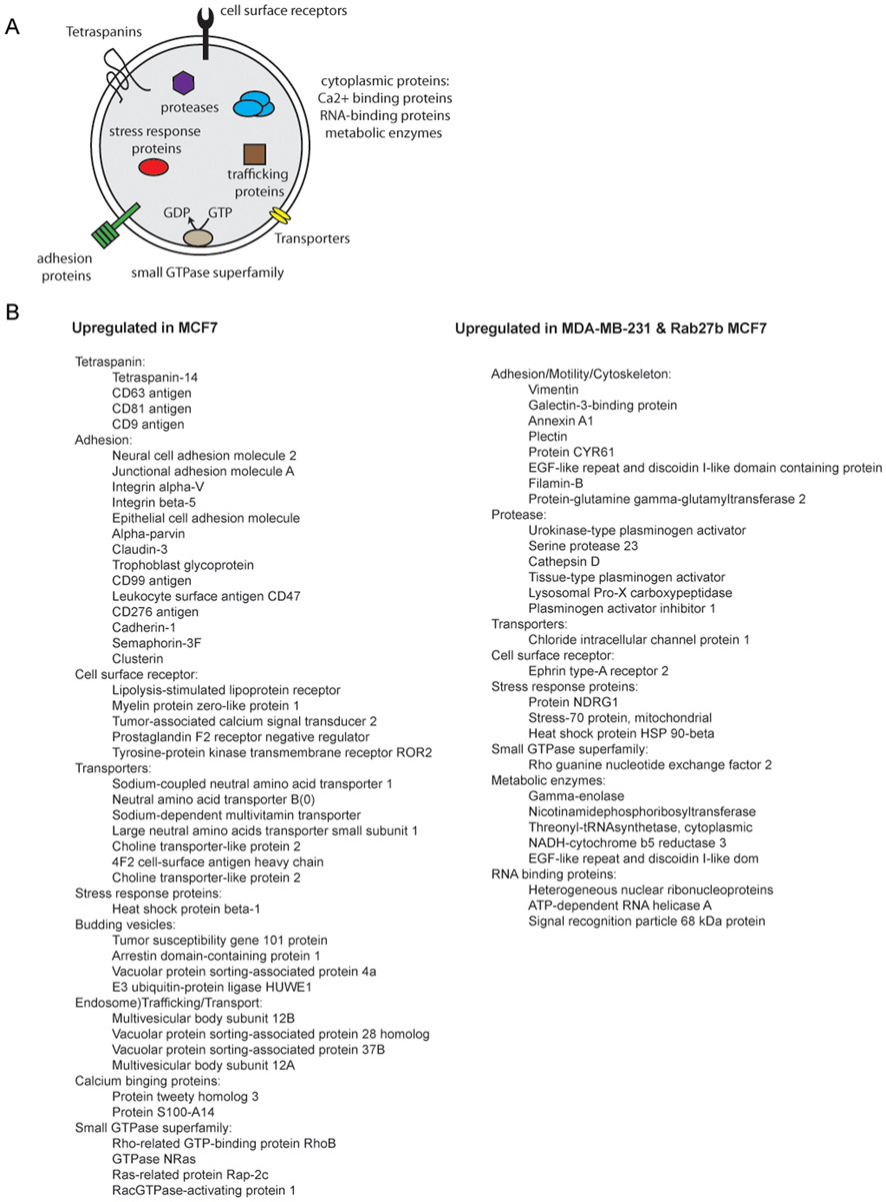
Schematic representation of exosome composition and differentially expressed tumor-derived proteins. (A) Cartoon of exosome with categories of identified associated proteins. (B) Table of differentially expressed proteins isolated from tumor-derived exosomes (MCF-7; Rab27b transformed MCF-7; MDA-MB-231) determined by iTRAQ analysis. The average ratio (≥2 fold) of differentially expressed exosomal proteins across MDA-MB-231/MCF-7 and Rab27b/MCF-7 were calculated and listed in the table.

The level of migration is likely due to the molecular composition of exosomes. Proteomic iTRAQ analysis of tumor-derived exosomal proteins allowed us to compare the expression of exosomal proteins from these three breast cancer cell lines. Our data provides a “metastatic signature” of these cells. 85 Differentially expressed proteins (~2-fold increase or decrease in relative expression) across the three breast cancer cell lines were found. Analysis of tumor-derived exosomes from the non-invasive breast cancer cell line MCF-7 revealed many up-regulated proteins commonly found in exosome preparations. Some of these proteins have known tumor suppressor function (Fig. 6). The catalogue of differentially enriched proteins in MCF-7-derived exosomes can be clustered into 9 structural/functional categories. The clusters obtained are: (1) tetraspanins; (2) adhesions; (3) calcium binding proteins; (4) cell surface receptors; (5) transporters; (6) small GTPase superfamily; (7) endosomal trafficking; (8) stress response proteins; and (9) vesicle budding. The remaining exosomal proteins (38/85 total; 45%) were identified as up-regulated more than 2-fold in the MDA-MB-231 and Rab27b cells. We observed a strong trend in expression patterns from these exosomes. In both lines, we observed significantly enriched populations of proteins with known functions in tumorigenesis and metastasis (Fig. 6).

Our results collectively identify the proteins and expression levels of exosomal proteins released from breast cancer cells under normal growth conditions. During metastasis, secreted proteins in the extracellular space are major factors in cell invasion, migration, motility, growth control, angiogenesis, matrix-degradation and adhesion [21]. An invasive role of tumor-derived exosomes has also been shown by work from Higginbotham *et*. *al* which identified exosomes derived from cells stably expressing amphiregulin, an epidermal growth factor family member, increased invasiveness of “recipient” breast cancer cells by five-fold [46]. Of the 85 differentially expressed proteins identified, 38 of them were up-regulated in MDA-MB-231 cells. More importantly, 30 of the 38 total (79%) up-regulated invasive exosomal proteins are also identified as potential cancer markers as overexpression of these identified proteins in cancer correlates with accelerated tumor growth, invasion, poor prognosis, and poor outcome in patients [24,26,27]. While we are unable to identify the specific molecules responsible for this effect, this approach should allow for targeted investigation of the proteins specifically up-regulated in these exosomes.

For example, we identified several members of the tetraspanins family (Tetraspanin-14, CD9, CD63, and CD81 antigens) to be increased in tumor-derived exosomes from the non-invasive cell line, MCF-7. Tetraspanins are implicated in a diverse range of biological phenomena, including cell motility, metastasis, cell proliferation and differentiation and are known to complex with integrins [47] [48,49]. Consistent with our iTRAQ analysis on tumor-derived exosomes, members of the tetraspanin superfamily of proteins are down-regulated in metastatic tumors, reduced [50] metastasis *in vivo*, and decrease cell migration [51–55]. Tetraspanin proteins involved in integrin-mediated cell adhesion at the plasma membrane and could control cell migration by regulating the connection between the cell and the extracellular matrix [56].

Many differentially expressed proteins in the exosomes belonged to the cluster of adhesion proteins. Unlike tetraspanins, these proteins show an increased expression pattern in metastatic exosomes. Extracellular matrix adhesion is critical in cancer biology and integrins are key components mediating this adhesion [55]. In the more metastatic tumor-derived exosomes, we observed up-regulation of unique set of adhesion proteins. These adhesion proteins induce changes in cell shape, motility, and adhesion. Adhesion molecules act as both positive and negative modulators of the metastatic process [57]. These various regulators of adhesion in the cells are represented in the composition of adhesion proteins in tumor-derived exosomes from breast cancer cells of varying invasiveness.

We show that there are a relatively small number of proteins that are differentially expressed in exosomes from all three breast cancer cell lines. Many of these proteins have been shown to have known functions in migration. A similar observation was made by difference, microarray, and proteomics analysis of breast cancer cells of varying invasiveness [26]. Their finding showed that a small subset of proteins may serve as an accurate signature of metastasis. We propose that there is likewise a unique pattern of expressed proteins contained in tumor-derived exosomes. This signature can be used to define the metastatic potential of the cell that released the exosomes. Further studies will be required to determine if these proteins are therapeutic targets.

## METHODS

### Constructs and transfections

EGFP-Rab27b plasmid was kindly provided by Dr. Wendy Westbroek (NHGRI, NIH, Bethesda, USA). Breast cancer cell lines MCF-7 and MDA-MB-231 were stably or transiently transfected using Lipofectamine 2000 (Invitrogen). To establish stable cell lines, singly transfected cells were selected in G418 (1mg/ml) (Invitrogen) for >4 weeks.

### Cell culture

The non-invasive/non-metastatic breast cancer cell line, MCF-7 and invasive/metastatic breast cancer cell line, MDA-MB-231 (ATCC) was cultured in Dulbecco’s modified eagles medium (DMEM; Gibco) supplemented with 10% fetal bovine serum (FBS; Atlanta Biologicals), 100U/ml penicillin, and 100μg/ml streptomycin, in a 5% CO2 humidified atmosphere at 37°C. For exosome purification, cells were cultured until ~90% confluency (~7–8 days) with DMEM and FBS deprived of bovine microvesicles by ultracentrifugation (4 hours at 200,000 x *g*). Cells were plated onto collagen-coated coverslips.

### Isolation tumor-derived exosomes

Exosomes were purified by differential centrifugation as previously described with slight modifications to the ultracentrifugation speeds and times [38],[41,58]. Briefly, culture medium containing exosome-free FBS from cells were collected after ~90% confluency, filtered by 0.22μm syringe filter to exclude cell debris, ultrafiltrated and condensed through Amico Ultra-15 centrifugal (Millipore) concentrators. The condensed culture medium was centrifuged at 2,000 x *g* for 10 minutes, followed by 21,000 x *g* for 30 minutes using a type SS-34 rotor in a Sorvall Evolution RC centrifuge (Beckman Coulter, USA), and then 200,000 x *g* for 2 hours using a type 70 Ti rotor in a Beckman Coulter Optima L-100 XP utracentrifuge (Beckman Coulter, USA) to remove exosomes further. The exosome pellet was resuspended in PBS.

### Fluorescent labeling of exosomes

For labeling exosome proteins, 500μg exosome protein was added to 1ml 0.1M sodium bicarbonate buffer (pH ~8.3) containing 100μg TAMRA-NHS (carboxytetramethylrhodaminesuccinimidyl ester, Biotium). Reactions were performed for 1 hour at room temperature. The unincorporated TAMRA-NHS was removed by using a 100kDa ultrafiltration tube (Millipore) and ten 30 minutes extended washing in PBS before concentrate was collected and washed once in PBS with 200,000 x *g* centrifugation for 1 hour. The concentrated solution was collected, diluted to 200μl in PBS, and used directly for exosome uptake assays. For the purposes of removing unincorporated TAMRA, we compared purification of TAMRA-labeled exosomes using ultrafiltration (100kDA ultrafiltration tube (Millipore)), dialysis (10kDa slide-a-lyzer Dialysis Cassettes) and PD-10 desalting column. All the purifications work equally well and give similar results for exosome uptake assays (data not shown).

### Electron microscopy

Five microliters aliquots of resuspended exosomes were placed onto support grids (Cu 200M), and allowed to absorb for 60 seconds. Grids were washed twice in double distilled water and vesicles were then stained for 10 sec in 2% uranyl acetate. The size and morphology of the particles were examined using a transmission electron microscope.

### Exosome uptake assay

Fluorescently labeled, TAMRA-exosomes (10–50μlu derived from breast cancer cells (MCF-7; Rab27b; MDA-MB-231) were added to recipient cells and monitored using EPI fluorescence. Excessive exosomes were washed by culture media at various time intervals. To quantify the cellular uptake of exosomes and for each experiment more than 50 cells were imaged. All the settings of imaging and processing were kept constant and the relative fluorescent intensities were calculated.

### Wound healing assay

MCF-7, Rab27b-transformed MCF-7, or MDA-MB-231 breast cancer cells were plated on collagen coverslips, into culture inserts (Idibi) specially made for wound healing assays. Wound healing assay were performed as previously described [59,60]. Briefly, attached cells were washed twice with 1x PBS and serum-free media (SFM) was used to cover each coverslip along with control, PBS, or 50μg exosomes isolated from the three breast cancer cell lines. Pictures were taken immediately after wound closure (15–21 hours depending on cell types).

### Confocal microscopy

Images were captured with a Zeiss LSM 510 confocal microscope with 488nm and 561nm lasers and a 63x objective lens. Filters used were 500–530 nm (EGFP) and 550–650nm (mCherry).

### Proteomics/Liquid Chromatography-Coupled Tandem Mass Spectrometry

Eluted proteins were reduced and alkylated in solution using iodoacetamide, and proteolyzed with sequencing grade modified porcine trypsin (Promega, Madison, WI). Protein identification by LC-MS/MS analysis of peptides was performed with an LTQ ion trap MS (Thermo Fisher Scientific, San Jose, CA) interfaced with a 2D nanoLC system (Eksigent, Dublin, CA). Peptides were fractionated by reverse-phase HPLC on a 75 μm×100 mm C18 column with a 10 μm emitter using a 0–60% acetonitrile/0.5% formic acid gradient over 30 min at 300 nl/min. Peptide sequences were identified using Mascot software (version 2.2, Matrix Science, Boston, MA) to search the NCBI non-redundant database with the acquired fragmentation data. Identified sequences were confirmed manually by inspecting the fragmentation spectra. Scaffold (version Scaffold_2_04_00, Proteome Software Inc., Portland, OR) was used to validate MS/MS based peptide and protein identifications. Peptide identifications were accepted if they could be established at greater than 95% probability as specified by the Peptide Prophet algorithm embedded within Scaffold. Low probability enabled potentially significant protein identifications based on many moderate-probability peptides, but no such proteins emerged; instead nearly all identifications were based on peptides with individual confidences >80%. Protein identifications were accepted if they could be established at greater than 89% probability based on at least 1 identified peptide. Protein probabilities were assigned by the embedded Protein Prophet algorithm. Proteins that contained similar peptides and could not be differentiated based on MS/MS analysis alone were grouped to satisfy the principles of parsimony.

### iTRAQ Quantitative proteomics

Exosomes were lysed in lysis buffer (2% SDS, 1% Triton-X100, 0.1M Tris pH 7.4, 1 tablet Complete EDTA-free protease inhibitors (Roche, Indianapolis, IN, USA) and concentrations were determined using BSA protein assay (Pierce, Rockford, IL, USA)). Exosome samples derived from MCF-7, Rab27b, MDA-MB-231 breast cancer cells (100μg each) were labeled with iTRAQ (isobaric tagging for relative and absolute quantification) reagents (Life Technologies), purified and fractionated with strong cation exchange chromatography (SCX) and analyzed on a VelosOrbitrap mass spectrometer. After proteins were precipitated, samples were mixed with 6 volumes of cold acetone and incubated for 60 min at -20°C. After 10 min of centrifugation at 13,000x *g* at 4°C, the supernatant was removed and the rest of the acetone was allowed to evaporate from the uncapped tubes at room temperature. Next, proteins were digested by reconstituting protein pellets with 20:l of dissolution buffer (0.5M triethylammonium bicarbonate), 1:l denaturant (2% SDS), and 2:l reducing reagent [50mM tris-(2-carboxyethyl) phosphine]. The sample were mixed by vortexing, spun down and incubated at 60°C for 1 hour. Free cysteines were blocked by adding 1:l of 200μM methyl methanethiosulfonate in isopropanol and incubated for 10 min at room temperature. Trypsin (Promega) was reconstituted with deionized water at 1μg/μl concentration. Trypsin (10μl) solution was added to each vial and incubated overnight at 37°C followed by iTRAQ labeling. 8-Plex iTRAQ reagents were allowed to reach room temperature and then reconstituted with 50ml of isopropanol. Each label reagent was mixed with the corresponding protein digest and incubated at room temperature for 2 hours. Samples were pooled into a new vial and dried using a centrifugal evaporator (Speedvac). After reconstituted with 0.1% formic acid (FA), the digest was desalted on an Oasis HLB 1 cc column (Waters, Milford, MA, USA) and eluted with 60% acetonitrile (ACN) 0.1% FA. Eluted peptide mixtures were dried by centrifugation evaporation, reconstituted with 100μl SCX buffer A (10mM KH_2_PO_4_, 20% ACN, pH 2.7) and separated on a PolyLCPolySULFOETHYL A column (200x 2.1 m, 5 mm, 200 Å) with a linear 200μl/min gradient of 0–70% buffer B (10mM KH_2_PO_4_, 20% ACN, 500mMKCl, pH 2.7) in 45 min on an Agilent 1200 LC device with Chemstation B.02.01 control software (Agilent, Santa Clara, CA, USA). Fractions were collected each minute and eventually pooled into 24 fractions. After vacuum centrifugation to evaporate the solute, fractions were desalted, eluted, dried as described above and reconstituted with 0.1% FA. Liquid chromatography was performed on an Eksigent nanoLC-Ultra 1D plus system (Eksigent, Dublin, CA, USA). Peptide digest was first loaded on a Zorbax 300SB-C18 trap (Agilent) at 6μl/min for 5 min, then separated on a PicoFrit analytical column (100mm long, ID 75μm, tip ID 10μm, packed with BetaBasic 5μm 300 Å particles; New Objective, Woburn, MA, USA) using a 40-min linear gradient of 5–35% ACN in 0.1% FA at a flow rate of 250 nl/min. Mass analysis was carried out on an LTQ OrbitrapVelos (Thermo Fisher Scientific, San Jose, CA, USA) with data-dependent analysis mode, where MS1 scanned full MS mass range from m/z 300 to 2,000 at 30,000 mass resolution and 6 HCD MS2 scans were sequentially carried out at resolution of 7,500 with 45% collision energy, both in the Orbitrap.

### Database search and quantitative data analysis

MS/MS spectra from 24 fractions were searched against the Swiss Prot (Swiss Institute of Bioinformatics, updated August 10, 2010, 21,241 entries) database, taxonomy Human using our 6-processor Mascot (Matrix Science, London, UK; version 2.3) cluster at NIH (http://biospec.nih.gov), with precursor mass tolerance at 20 ppm, fragment ion mass tolerance at 0.05 Da, trypsin enzyme with 2 miscleavages, methyl methanethiosulfonate of cysteine and iTRAQ 8plex of lysine and the N-terminus as fixed modifications, and deamidation of asparagine and glutamine, oxidation of methionine and iTRAQ 8plex of tyrosine as variable modifications. The resulting data file was load into Scaffold Q_(version Scaffold_3_00_04, Proteome Software Inc., Portland, OR, USA) to filter and quantitate peptides and proteins. Peptide identifications were accepted at 95.0% or higher probability as specified by the Peptide Prophet algorithm and a false discovery rate (FDR) of less than 1%. Protein identifications were accepted at 99.0% or higher probability and contained at least 2 identified peptides with FDR less than 1%. Protein probabilities were assigned by the Protein Prophet algorithm. Proteins that contained similar peptides and could not be differentiated based on MS/MS analysis alone were grouped to satisfy the principles of parsimony. Peptides were quantitated using the centroid reporter ion peak intensity, with minimum of 5% of the highest peak in the spectrum. Intrasample channels were normalized based on the median ratio for each channel across all proteins. The isobaric tagged samples were normalized by comparing the median protein ratios for the reference channel. Quantitative protein values were derived from only uniquely assigned peptides. The proteomic data has been submitted to the Vesiclepedia database [61], http://www.microvesicles.org/.

### Western blotting

Cells were washed 3x with PBS (Life Technologies, Grand Island, NY) and scraped and lysed in RIPA buffer containing a protease inhibitor mix (Roche Diagnostics, Indianapolis, IN). The lysates were sonicated, mixed with 2x Laemmli sample buffer (Bio-Rad, Hercules, CA)in a 1:1 ratio and boiled for 5 min followed by centrifugation at 5,000g for 10 min. 20 microgram of total protein, as determined by DC protein assay (Bio-Rad), was loaded onto 4–20% Criterion TGX precast gels (Bio-Rad). The Trans-Blot Turbo (Bio-Rad) device was used for transfer onto PVDF membranes (Bio-Rad). Membranes were dried followed by activation in 100% methanol and blocking in PBS (Life technologies) plus 5% milk and 0.5% Tween (Sigma, St. Louis, MO) for 60 min. at RT. The protein containing PVDF membranes were probed overnight in PBS with 5% milk and 0.5% Tween at 4°C with a mouse monoclonal antibody demonstrated to be specific for Rab27A [37] (BD Biosciences, San Jose, CA), an anti-Rab27B polyclonal antibody (Novus Biologicals, Littleton, CO), and an anti-GFP mouse monoclonal antibody (Millipore, Billerica, MA). An anti-β-Actin-HRP (Abcam, Cambridge, MA) antibody was used as a protein loading control. Membranes were washed 3x with PBS with 5% milk and 0.5% Tween for 5 min followed by incubation with secondary antibodies in PBS with 5% milk and 0.5% Tween at RT for 60 min. Membranes were washed 5x in PBS with 0.5% Tween for 5 min followed by ECL detection (Amersham Biosciences, Piscataway, NJ). For WB on purified exosomes 15 microgram of total protein was loaded; PVDF membranes were incubated with anti-Alix (Cell Signaling, Danvers, MA), anti Tsg101 (Santa Cruz Biotechnology Inc, Dallas, TX), anti-HSP90 (Thermo Scientific, West Palm Beach, FL), and anti-HSP70 (System Biosciences, Mountain View, CA). Secondary antibodies were HRP-conjugated secondary anti-mouse or anti-rabbit antibodies (Amersham Biosciences, Piscataway, NJ).

### Nanoparticle Tracking Analysis

The size and concentration of particles were determined via nanoparticle tracking analysis (NTA) using a NanoSight LM10-HS instrument (NanoSight, Amesbury, UK) equipped with a 405nm laser. 5 μg protein was diluted in 1.5 ml PBS. For each condition three independent samples were prepared and batch processed. Per measurement a movie of 60 s was recorded. All data were analyzed with the NTA Analytical Software suite version 2.3. The NanoSight system was calibrated with polystyrene latex microbeads (Thermo Scientific) prior to analysis.

### Matrigel Invasion assay

For each cell line, 1x10^5^ cells were seeded in transwell chambers with Matrigel-coated membrane (24-well insert; pore size 8 μm; Becton Dickinson, Franklin Lakes, NJ) in serum-and antibiotic-free DMEM medium; DMEM with 10% fetal calf serum was used as chemoattractant in the lower chamber. Cells that did not invade through the pores of the membrane were removed with a cotton swab after 48 hours. The membrane was mounted on a coverslip and cells on the lower surface of the membrane were stained with ProLog Gold antifade reagent with DAPI (Life Technologies, Grand Island, NY). Invasive cells were counted in 10 microscopic fields using a fluorescence microscope (Axiovert 200M; Carl Zeiss, Thornwood, NY) with a 40× objective. Statistical analyses were performed using GraphPadPrism5.0 software. Matrigel invasion data were analyzed by using two-sided Student *t* tests.

## Supporting Information

S1 Fig Nanoparticle tracking analysis (NTA) of tumor-derived exosomes/microvesicles. NTA of exosome/microvesicles derived from (A) MCF-7; (B) MCF-7/Rab27b; and (C) MDA-MB-231. Size and particle distribution (concentration) plots of exosome samples from all three cells lines done in independent and duplicate preparations of each cell line.
(TIF)

S1 Table Proteomics and iTRAQ analysis of tumor derived exosomes/microvesicles. (1) Identification of MCF-7 tumor-derived exosomes from in-gel digestion; (2) iTRAQ analysis of tumor-derived exosomal proteins from MCF-7; Rab27b; and MDA-MB-231 breast cancer cells lines.
(XLSX)

S2 Table Proteomics and iTRAQ analysis of tumor derived exosomes/microvesicles. (1) Identification of MCF-7 tumor-derived exosomes from in-gel digestion; (2) iTRAQ analysis of tumor-derived exosomal proteins from MCF-7; Rab27b; and MDA-MB-231 breast cancer cells lines.
(XLSX)

## References

[1] Thery C, Zitvogel L, Amigorena S (2002) Exosomes: composition, biogenesis and function. Nat Rev Immunol 2: 569–579. 12154376 10.1038/nri855

[2] Iero M, Valenti R, Huber V, Filipazzi P, Parmiani G, et al (2008) Tumour-released exosomes and their implications in cancer immunity. Cell Death Differ 15: 80–88. 17932500 10.1038/sj.cdd.4402237

[3] Ratajczak J, Wysoczynski M, Hayek F, Janowska-Wieczorek A, Ratajczak MZ (2006) Membrane-derived microvesicles: important and underappreciated mediators of cell-to-cell communication. Leukemia 20: 1487–1495. 16791265 10.1038/sj.leu.2404296

[4] Cocucci E, Racchetti G, Meldolesi J (2009) Shedding microvesicles: artefacts no more. Trends Cell Biol 19: 43–51. 10.1016/j.tcb.2008.11.003 19144520

[5] Valadi H, Ekstrom K, Bossios A, Sjostrand M, Lee JJ, et al (2007) Exosome-mediated transfer of mRNAs and microRNAs is a novel mechanism of genetic exchange between cells. Nat Cell Biol 9: 654–659. 17486113 10.1038/ncb1596

[6] Peinado H, Lavotshkin S, Lyden D (2011) The secreted factors responsible for pre-metastatic niche formation: old sayings and new thoughts. Semin Cancer Biol 21: 139–146. 10.1016/j.semcancer.2011.01.002 21251983

[7] Ratajczak J, Miekus K, Kucia M, Zhang J, Reca R, et al (2006) Embryonic stem cell-derived microvesicles reprogram hematopoietic progenitors: evidence for horizontal transfer of mRNA and protein delivery. Leukemia 20: 847–856. 16453000 10.1038/sj.leu.2404132

[8] Nazarenko I, Rana S, Baumann A, McAlear J, Hellwig A, et al (2010) Cell surface tetraspanin Tspan8 contributes to molecular pathways of exosome-induced endothelial cell activation. Cancer Res 70: 1668–1678. 10.1158/0008-5472.CAN-09-2470 20124479

[9] Webber J, Steadman R, Mason MD, Tabi Z, Clayton A (2010) Cancer exosomes trigger fibroblast to myofibroblast differentiation. Cancer Res 70: 9621–9630. 10.1158/0008-5472.CAN-10-1722 21098712

[10] Ginestra A, La Placa MD, Saladino F, Cassara D, Nagase H, et al (1998) The amount and proteolytic content of vesicles shed by human cancer cell lines correlates with their in vitro invasiveness. Anticancer Res 18: 3433–3437. 9858920

[11] Ginestra A, Miceli D, Dolo V, Romano FM, Vittorelli ML (1999) Membrane vesicles in ovarian cancer fluids: a new potential marker. Anticancer Res 19: 3439–3445. 10629632

[12] Camussi G, Deregibus MC, Bruno S, Grange C, Fonsato V, et al (2011) Exosome/microvesicle-mediated epigenetic reprogramming of cells. Am J Cancer Res 1: 98–110. 21969178 PMC3180104

[13] Schorey JS, Bhatnagar S (2008) Exosome function: from tumor immunology to pathogen biology. Traffic 9: 871–881. 10.1111/j.1600-0854.2008.00734.x 18331451 PMC3636814

[14] Castellana D, Toti F, Freyssinet JM (2010) Membrane microvesicles: macromessengers in cancer disease and progression. Thromb Res 125 Suppl 2: S84–88. 10.1016/S0049-3848(10)70021-9 20434014

[15] Peinado H, Aleckovic M, Lavotshkin S, Matei I, Costa-Silva B, et al (2012) Melanoma exosomes educate bone marrow progenitor cells toward a pro-metastatic phenotype through MET. Nat Med 18: 883–891. 10.1038/nm.2753 22635005 PMC3645291

[16] Hao S, Ye Z, Li F, Meng Q, Qureshi M, et al (2006) Epigenetic transfer of metastatic activity by uptake of highly metastatic B16 melanoma cell-released exosomes. Exp Oncol 28: 126–131. 16837903

[17] Skog J, Wurdinger T, van Rijn S, Meijer DH, Gainche L, et al (2008) Glioblastoma microvesicles transport RNA and proteins that promote tumour growth and provide diagnostic biomarkers. Nat Cell Biol 10: 1470–1476. 10.1038/ncb1800 19011622 PMC3423894

[18] Hood JL, San RS, Wickline SA (2011) Exosomes released by melanoma cells prepare sentinel lymph nodes for tumor metastasis. Cancer Res 71: 3792–3801. 10.1158/0008-5472.CAN-10-4455 21478294

[19] Mineo M, Garfield SH, Taverna S, Flugy A, De Leo G, et al (2011) Exosomes released by K562 chronic myeloid leukemia cells promote angiogenesis in a Src-dependent fashion. Angiogenesis 15: 33–45. 10.1007/s10456-011-9241-1 22203239 PMC3595015

[20] Park JE, Tan HS, Datta A, Lai RC, Zhang H, et al (2010) Hypoxic tumor cell modulates its microenvironment to enhance angiogenic and metastatic potential by secretion of proteins and exosomes. Mol Cell Proteomics 9: 1085–1099. 10.1074/mcp.M900381-MCP200 20124223 PMC2877972

[21] Sethi N, Kang Y (2011) Unravelling the complexity of metastasis—molecular understanding and targeted therapies. Nat Rev Cancer 11: 735–748. 10.1038/nrc3125 21941285 PMC10961136

[22] Frost AR, Hurst DR, Shevde LA, Samant RS (2012) The influence of the cancer microenvironment on the process of metastasis. Int J Breast Cancer 2012: 756257 10.1155/2012/756257 22570792 PMC3337591

[23] Brabek J, Mierke CT, Rosel D, Vesely P, Fabry B (2010) The role of the tissue microenvironment in the regulation of cancer cell motility and invasion. Cell Commun Signal 8: 22 10.1186/1478-811X-8-22 20822526 PMC2941745

[24] Ramaswamy S, Ross KN, Lander ES, Golub TR (2003) A molecular signature of metastasis in primary solid tumors. Nat Genet 33: 49–54. 12469122 10.1038/ng1060

[25] Roepman P, de Jager A, Groot Koerkamp MJ, Kummer JA, Slootweg PJ, et al (2006) Maintenance of head and neck tumor gene expression profiles upon lymph node metastasis. Cancer Res 66: 11110–11114. 17145852 10.1158/0008-5472.CAN-06-3161

[26] Nagaraja GM, Othman M, Fox BP, Alsaber R, Pellegrino CM, et al (2006) Gene expression signatures and biomarkers of noninvasive and invasive breast cancer cells: comprehensive profiles by representational difference analysis, microarrays and proteomics. Oncogene 25: 2328–2338. 16314837 10.1038/sj.onc.1209265

[27] Lu Y, Yi Y, Liu P, Wen W, James M, et al (2007) Common human cancer genes discovered by integrated gene-expression analysis. PLoS One 2: e1149 17989776 10.1371/journal.pone.0001149 PMC2065803

[28] Pierre M, DeHertogh B, Gaigneaux A, DeMeulder B, Berger F, et al (2010) Meta-analysis of archived DNA microarrays identifies genes regulated by hypoxia and involved in a metastatic phenotype in cancer cells. BMC Cancer 10: 176 10.1186/1471-2407-10-176 20433688 PMC2880990

[29] Bobrie A, Krumeich S, Reyal F, Recchi C, Moita LF, et al (2012) Rab27a supports exosome-dependent and-independent mechanisms that modify the tumor microenvironment and can promote tumor progression. Cancer Res 72: 4920–4930. 10.1158/0008-5472.CAN-12-0925 22865453

[30] Hessvik NP, Phuyal S, Brech A, Sandvig K, Llorente A (2012) Profiling of microRNAs in exosomes released from PC-3 prostate cancer cells. Biochim Biophys Acta 1819: 1154–1163. 10.1016/j.bbagrm.2012.08.016 22982408

[31] Palazzolo G, Albanese NN, G DIC, Gygax D, Vittorelli ML, et al (2012) Proteomic analysis of exosome-like vesicles derived from breast cancer cells. Anticancer Res 32: 847–860. 22399603

[32] Lai TC, Chou HC, Chen YW, Lee TR, Chan HT, et al (2014) Secretomic and proteomic analysis of potential breast cancer markers by two-dimensional differential gel electrophoresis. J Proteome Res 9: 1302–1322. 10.1021/pr900825t 20052998

[33] Simpson RJ, Lim JW, Moritz RL, Mathivanan S (2009) Exosomes: proteomic insights and diagnostic potential. Expert Rev Proteomics 6: 267–283. 10.1586/epr.09.17 19489699

[34] Simpson RJ, Jensen SS, Lim JW (2008) Proteomic profiling of exosomes: current perspectives. Proteomics 8: 4083–4099. 10.1002/pmic.200800109 18780348

[35] Turner S, Sherratt JA (2002) Intercellular adhesion and cancer invasion: a discrete simulation using the extended Potts model. J Theor Biol 216: 85–100. 12076130 10.1006/jtbi.2001.2522

[36] Neve RM, Chin K, Fridlyand J, Yeh J, Baehner FL, et al (2006) A collection of breast cancer cell lines for the study of functionally distinct cancer subtypes. Cancer Cell 10: 515–527. 17157791 10.1016/j.ccr.2006.10.008 PMC2730521

[37] Hendrix A, Maynard D, Pauwels P, Braems G, Denys H, et al (2010) Effect of the secretory small GTPase Rab27B on breast cancer growth, invasion, and metastasis. J Natl Cancer Inst 102: 866–880. 10.1093/jnci/djq153 20484105 PMC2886092

[38] Thery C, Amigorena S, Raposo G, Clayton A (2006) Isolation and characterization of exosomes from cell culture supernatants and biological fluids Curr Protoc Cell Biol Chapter 3: Unit 3 22. 10.1002/0471143030.cb0322s30 18228490

[39] Bobrie A, Colombo M, Krumeich S, Raposo G, Thery C (2012) Diverse subpopulations of vesicles secreted by different intracellular mechanisms are present in exosome preparations obtained by differential ultracentrifugation. J Extracell Vesicles 1 10.3402/jev.v1i0.19179 24009879 PMC3760636

[40] Thery C, Ostrowski M, Segura E (2009) Membrane vesicles as conveyors of immune responses. Nat Rev Immunol 9: 581–593. 10.1038/nri2567 19498381

[41] Tian T, Wang Y, Wang H, Zhu Z, Xiao Z (2010) Visualizing of the cellular uptake and intracellular trafficking of exosomes by live-cell microscopy. J Cell Biochem 111: 488–496. 10.1002/jcb.22733 20533300

[42] Dimmer EC, Huntley RP, Alam-Faruque Y, Sawford T, O'Donovan C, et al (2011) The UniProt-GO Annotation database in 2011. Nucleic Acids Res 40: D565–570. 10.1093/nar/gkr1048 22123736 PMC3245010

[43] Muralidharan-Chari V, Clancy JW, Sedgwick A, D'Souza-Schorey C (2010) Microvesicles: mediators of extracellular communication during cancer progression. J Cell Sci 123: 1603–1611. 10.1242/jcs.064386 20445011 PMC2864708

[44] Chaerkady R, Harsha HC, Nalli A, Gucek M, Vivekanandan P, et al (2008) A quantitative proteomic approach for identification of potential biomarkers in hepatocellular carcinoma. J Proteome Res 7: 4289–4298. 10.1021/pr800197z 18715028 PMC3769105

[45] Koumangoye RB, Sakwe AM, Goodwin JS, Patel T, Ochieng J (2011) Detachment of breast tumor cells induces rapid secretion of exosomes which subsequently mediate cellular adhesion and spreading. PLoS One 6: e24234 10.1371/journal.pone.0024234 21915303 PMC3167827

[46] Higginbotham JN, Demory Beckler M, Gephart JD, Franklin JL, Bogatcheva G, et al (2011) Amphiregulin exosomes increase cancer cell invasion. Curr Biol 21: 779–786. 10.1016/j.cub.2011.03.043 21514161 PMC3417320

[47] Berditchevski F (2001) Complexes of tetraspanins with integrins: more than meets the eye. J Cell Sci 114: 4143–4151. 11739647 10.1242/jcs.114.23.4143

[48] Liu L, He B, Liu WM, Zhou D, Cox JV, et al (2007) Tetraspanin CD151 promotes cell migration by regulating integrin trafficking. J Biol Chem 282: 31631–31642. 17716972 10.1074/jbc.M701165200

[49] Mantegazza AR, Barrio MM, Moutel S, Bover L, Weck M, et al (2004) CD63 tetraspanin slows down cell migration and translocates to the endosomal-lysosomal-MIICs route after extracellular stimuli in human immature dendritic cells. Blood 104: 1183–1190. 15130945 10.1182/blood-2004-01-0104

[50] Pelchen-Matthews A, Raposo G, Marsh M (2004) Endosomes, exosomes and Trojan viruses. Trends Microbiol 12: 310–316. 15223058 10.1016/j.tim.2004.05.004

[51] Boucheix C, Duc GH, Jasmin C, Rubinstein E (2001) Tetraspanins and malignancy. Expert Rev Mol Med 2001: 1–17. 14987371 10.1017/S1462399401002381

[52] Guo XZ, Friess H, Di Mola FF, Heinicke JM, Abou-Shady M, et al (1998) KAI1, a new metastasis suppressor gene, is reduced in metastatic hepatocellular carcinoma. Hepatology 28: 1481–1488. 9828210 10.1002/hep.510280606

[53] Ikeyama S, Koyama M, Yamaoko M, Sasada R, Miyake M (1993) Suppression of cell motility and metastasis by transfection with human motility-related protein (MRP-1/CD9) DNA. J Exp Med 177: 1231–1237. 8478605 10.1084/jem.177.5.1231 PMC2191011

[54] Radford KJ, Mallesch J, Hersey P (1995) Suppression of human melanoma cell growth and metastasis by the melanoma-associated antigen CD63 (ME491). Int J Cancer 62: 631–635. 7665237 10.1002/ijc.2910620523

[55] Dong JT, Lamb PW, Rinker-Schaeffer CW, Vukanovic J, Ichikawa T, et al (1995) KAI1, a metastasis suppressor gene for prostate cancer on human chromosome 11p11.2. Science 268: 884–886. 7754374 10.1126/science.7754374

[56] Shen X, Li CC, Aponte AM, Shen RF, Billings EM, et al (2012) Brefeldin A-inhibited ADP-ribosylation factor activator BIG2 regulates cell migration via integrin beta1 cycling and actin remodeling. Proc Natl Acad Sci U S A 109: 14464–14469. 10.1073/pnas.1211877109 22908276 PMC3437864

[57] Zetter BR (1993) Adhesion molecules in tumor metastasis. Semin Cancer Biol 4: 219–229. 8400144

[58] Ochieng J, Pratap S, Khatua AK, Sakwe AM (2009) Anchorage-independent growth of breast carcinoma cells is mediated by serum exosomes. Exp Cell Res 315: 1875–1888. 10.1016/j.yexcr.2009.03.010 19327352 PMC2742412

[59] McCready J, Sims JD, Chan D, Jay DG (2010) Secretion of extracellular hsp90alpha via exosomes increases cancer cell motility: a role for plasminogen activation. BMC Cancer 10: 294 10.1186/1471-2407-10-294 20553606 PMC3087318

[60] O'Brien K, Rani S, Corcoran C, Wallace R, Hughes L, et al (2013) Exosomes from triple-negative breast cancer cells can transfer phenotypic traits representing their cells of origin to secondary cells. Eur J Cancer 49: 1845–1859. 10.1016/j.ejca.2013.01.017 23453937

[61] Kalra H, Simpson RJ, Ji H, Aikawa E, Altevogt P, et al (2012) Vesiclepedia: a compendium for extracellular vesicles with continuous community annotation. PLoS Biol 10: e1001450 10.1371/journal.pbio.1001450 23271954 PMC3525526

[62] Abdelkarim M, Vintonenko N, Starzec A, Robles A, Aubert J, et al (2011) Invading basement membrane matrix is sufficient for MDA-MB-231 breast cancer cells to develop a stable in vivo metastatic phenotype. PLoS One 6: e23334 10.1371/journal.pone.0023334 21858074 PMC3156115

