# Supplementary figures and images for: Exosomes Released from Breast Cancer Carcinomas Stimulate Cell Movement
Source: PLoS One. 2015 Mar 23;10(3):e0117495. doi: 10.1371/journal.pone.0117495 (PMC4370373; doi:10.1371/journal.pone.0117495)

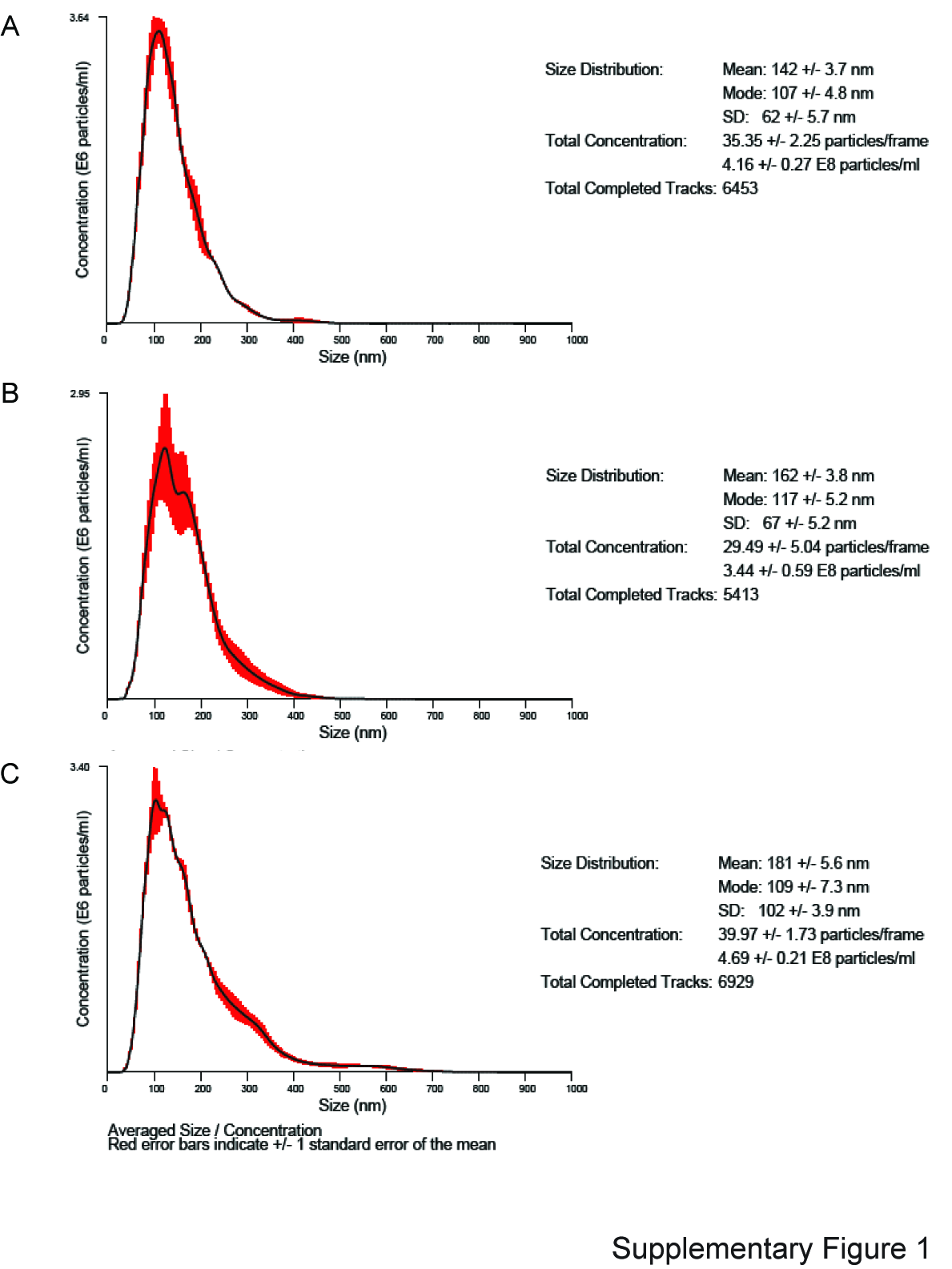

Supplement: S1 Fig — NTA of exosome/microvesicles derived from (A) MCF-7; (B) MCF-7/Rab27b; and (C) MDA-MB-231. Size and particle distribution (concentration) plots of exosome samples from all three cells lines done in independent and duplicate preparations of each cell line. (TIF) [file pone.0117495.s001.tif]
